# Non-invasive prenatal testing (NIPT): is routinization problematic?

**DOI:** 10.1186/s12910-023-00970-5

**Published:** 2023-10-26

**Authors:** Christoph Rehmann-Sutter, Daniëlle R. M. Timmermans, Aviad Raz

**Affiliations:** 1 Institute for History of Medicine and Science Studies, University of Lübeck, Lübeck, Germany; 2 Amsterdam UMC, Vrije Universiteit Amsterdam, Public and Occupational Health, Amsterdam Public Health Research Institute, Amsterdam, Netherlands; 3 Department of Sociology and Anthropology, Ben-Gurion University of the Negev, Be’er Sheva, Israel

**Keywords:** Routinization, Non-invasive prenatal testing, Prenatal diagnosis, Informed decision-making, Reproductive autonomy

## Abstract

**Background:**

The introduction and wide application of non-invasive prenatal testing (NIPT) has triggered further evolution of routines in the practice of prenatal diagnosis. ‘Routinization’ of prenatal diagnosis however has been associated with hampered informed choice and eugenic attitudes or outcomes. It is viewed, at least in some countries, with great suspicion in both bioethics and public discourse. However, it is a heterogeneous phenomenon that needs to be scrutinized in the wider context of social practices of reproductive genetics. In different countries with their different regulatory frameworks, different patterns of routines emerge that have different ethical implications.

*This paper* discusses an ethics of routines informed by the perspectives of organizational sociology and psychology, where a routine is defined as a repetitive, recognizable pattern of interdependent organizational actions that is carried out by multiple performers. We favour a process approach that debunks the view – which gives way to most of the concerns – that routines are always blindly performed. If this is so, routines are therefore not necessarily incompatible with responsible decision-making. Free and informed decision-making can, as we argue, be a key criterion for the ethical evaluation of testing routines. If free and informed decision-making by each pregnant woman is the objective, routines in prenatal testing may not be ethically problematic, but rather are defensible and helpful. We compare recent experiences of NIPT routines in the context of prenatal screening programmes in Germany, Israel and the Netherlands. Notable variation can be observed between these three countries (i) in the levels of routinization around NIPT, (ii) in the scope of routinization, and (iii) in public attitudes toward routinized prenatal testing.

**Conclusion:**

An ethics of routines in the field of prenatal diagnostics should incorporate and work with the necessary distinctions between levels and forms of routines, in order to develop sound criteria for their evaluation.

## Introduction

International bioethical literature and well-argued public concerns raised in many countries have suggested that the “routinization” of prenatal testing is ethically problematic, since it could hamper the quality and freedom of informed decision-making in each individual case, and increase social or moral pressure on women to test. Routine testing for Down syndrome or other relatively common conditions tested in NIPT could also add to stigmatization and discrimination of people who live with that condition. However, from a clinical perspective one can argue that prenatal care is inevitably a highly repetitive and hence routinized field of clinical practice and well-considered professional and clinical routines are essential parts of it. It is therefore hard to imagine how non-invasive prenatal testing (NIPT) could avoid being part of routines. It might rather depend on which forms of routines are adopted than whether routines happen to emerge at all.

It is well established in the sociological and anthropological literature that amniocentesis, maternal serum screening and diagnostic ultrasound have changed the experience of pregnancy and reconstructed the social meanings of motherhood [23, 41, 24, 25]. Ever since the introduction of prenatal screening tests, a possible “routinization” of testing and selective abortion was a matter of ethical concern [44]. While “high-risk” women were first included in testing, “low-risk” women – the vast majority of pregnancies – were subsequently addressed by large-scale screening programmes. NIPT, mainly designed for detecting a few common chromosomal anomalies of the foetus and some other genetic variants, were introduced to the screening toolbox and to clinical routines of many countries from 2011 onwards [34]. NIPT is even seen as one of relatively few examples of genomic medicine transforming routine clinical care [16], p. 460). Together with the estimation of the individual probability of giving birth to a child with Down syndrome, which provides information for individual decisions about the next step of a screening and testing chain, prenatal screening regimes, as some cautioned years before the introduction of NIPT, might ‘compromise’ the possibility of individual autonomous decision-making [32], p. 1002), while others [10] on the basis of a large qualitative study found that the active offer of an unsolicited prenatal test need not be considered as an impediment for making an autonomous choice, therefore, at least in principle, NIPT could also support autonomous decision making of the pregnant woman.

Since NIPT works with cell-free foetal DNA, which is present in maternal blood [30], it is not associated with any risk of iatrogenic pregnancy loss like invasive tests such as amniocentesis or chorionic villus sampling [1]. For women, one of the most important reasons to say no to prenatal testing – the potential for harm to her pregnancy, as low this risk may be – has disappeared.^1^ The discussion of routinization understandably became more intensive.

The 2017 report by the British Nuffield Council on Bioethics on ethical issues of NIPT mentions in a nuanced evaluation the establishment of routines as a main concern: “Some women accept screening because it is perceived to be a routine part of prenatal care, and in these cases it might be better described as an instance of conformity rather than active choice” (Nuffield [31], p. 10, Sect. 1.15). Also a more recent systematic review of potential concerns of pregnant people and clinicians on NIPT [54] found as a major concern of different involved groups that widening access to NIPT “may result in routinization of this test, causing potential harm to pregnant people, their families, the health care system, people living with disabilities, and society as a whole.” Adriana Kater-Kuipers et al. [19] conducted a systematic literature search especially on routinization of NIPT and found three specific groups of concerns about routinization: (a) *unconsidered choices*, because under a routine pregnant women do not deliberate and might not be aware of the consequences of testing; (b) *constrained freedom to choose*, since routine testing becomes self-evident (and will thus be used more frequently), and social pressure to take part in prenatal screening might be felt; (c) *negative consequences for people with disability*: acceptance of children with this condition might decline so that they then become more stigmatized and discriminated against. Hence, problems of adequate information, freedom of choice, and societal consequences are seen. The authors also deplore a significant lack of clarity about the meaning of the term ‘routinization’ in the literature [19], p. 630). The three areas of concern might indeed be important but, as we argue, not because they compromise prenatal screening routines per se but as criteria that can be used to evaluate different forms of routinization.

In order to see more clearly what the umbrella term of ‘routinization’ covers, and which ethical questions are the most pertinent to raise about routinization of NIPT, we first consider the state of research on routines in the sociology and psychology of organizations. We need to distinguish between different perspectives on routines and routine decisions (such as the pregnant woman’s or the healthcare provider’s), and how they are perceived from these perspectives, in order to see what is problematic and why.

We then look more closely at experiences of NIPT routines in the context of prenatal screening programmes in three countries. The different experiences of countries with different policies regarding the introduction of NIPT allow us to see which routines have emerged, how routines (as institutionalized arrangements) are being negotiated, created and deployed in the public health system to respond to a new technology (NIPT) which has disrupted earlier routines in the procedures of prenatal care. A comparison between Germany, Israel and the Netherlands provides evidence that current practices and discussions diverge in some key respects.

## Routinization: ethical, sociological, and psychological perspectives

Routines in prenatal care do not merely follow an instrumental logic and technological imperatives, but are also shaped by the cultural contexts of views on disability, eugenics, welfare and prevention. These views may lead both to demands for routines as an organizational requirement for efficiency and quality as well as to criticisms of problematic routinization.

The introduction of NIPT constitutes the most recent major transformation of prenatal diagnostic practices [25]. But NIPT is not a stand-alone technology, and hence it must be seen in the context of other procedures of obstetric care and genetic services. NIPT is today one of the most widely distributed “selective reproductive technologies” [55], developed against a backdrop of a “partly contingent coming together of three medical technologies – amniocentesis, the study of human chromosomes and obstetrical ultrasound – with a social innovation, the decriminalization of abortion,” which Ilana Löwy has called the “prenatal diagnosis dispositif” [22].

We focus here on the routinization of NIPT while it makes a transition from a second-tier test offered on a case-by-case basis in high-risk pregnancies, to a first-tier screening offered to all pregnant women, as it is the case in Belgium or the Netherlands [47, 2, 4]. This transition of NIPT into what should better be called NIPS (non-invasive prenatal *screening*) is perhaps no different in principle from the routinization of previous PND technologies such as biochemical or nuchal translucency screening. The significance of looking at NIPT/NIPS stems from examining the multiple social factors that shape a new and highly acclaimed biomedical technology, and the social impact of this genomic technology on patients and families as it makes the transition from testing into screening while still manifesting uncertainties and conflicts. Because this transition is still in the process of unfolding, with various questions (at which stage of pregnancy, under which conditions etc.) still being contested, NIPT/NIPS highlights a significant ethical dilemma of prenatal care in post-industrial societies: the routinization of prenatal screening for genetic risks within a normative framework that ought to be based on two ethical values [17]: (i) free choice and autonomy, (ii) respect for people with disabilities.

## Ethical perspective

Routinization, in the ethical literature discussing PND, often (but not exclusively) means a practice without deliberation, or without reflection, which therefore may contain an uninformed, perhaps involuntary decision. Like the concept of ‘medicalization’, which is often used critically, actually to indicate a kind of over-medicalization, the common use of ‘routinization’ also implies, by default, some problematic aspect. Routinization in this sensitive field would mean, as Susan Suter wrote in a classic paper, that “testing has become part of social norms and expectations” [44], p. 241), it is reflected “in attitudes of medical professionals, the patients offered prenatal testing and those they consult”, which results in a situation where women who *reject* such testing must be prepared to give explanations and justifications, whilst testing is the default. “People ask ‘Why not?’ No one asks ‘Why?’” (ibid.). One main bioethical concern has been that routinization represents *social pressure to conform to testing*, i.e. to comply with unwritten rules of a society. As already mentioned, the Nuffield Council ([31], Sect. 1.15) report on NIPT emphasizes that if NIPT is “perceived to be a routine part of prenatal care,” it might challenge the conveying of balanced and non-directive information to enable informed choice.

Giovanni Rubeis et al. ([42], p. 54) have proposed to define routinization in the practice of prenatal screening from an ethical point of view: a routine generally “signifies the use of a procedure as a *standard* measure.” Whether that means, as they claim, that the procedure in practice “is presented to patients as a *necessary* and *natural* element of prenatal care” (our emphasis) does however not seem to us to be fully evident. It would rather depend on the actual content and the form of the routine, i.e. *how* the procedure is routinely offered, and what kind of decision-making *situation* this routinely creates for the woman. We argue that routinization of NIPT does not therefore necessarily mean *applying* NIPT routinely, or even terminating a pregnancy routinely after a positive confirmatory test. Routines might also be preoccupied with creating a good decision-making situation *about* NIPT for the woman.

Empirical studies that have examined these concerns have shown that a great majority of women who underwent NIPT reported to have made an informed choice: 77,9% (of 1053 in the Netherlands; van Schendel et al. [52]) and in 76% (of 220 in England; Lewis et al. [20]). It is however unclear, which factors contribute to informed choice within routines, as well as whether and to what extent public attitudes towards Down syndrome will change if NIPT/NIPS is publicly perceived as routine. There is a need to distinguish between different elements and meanings of routinization. The provision of information and counselling is certainly one important element. In order to understand the phenomenon of routines more fully we can perhaps benefit from taking a step back and looking at how action and decision-making work within routines in the context of social practices and institutions.

## Sociological perspective

Originally, routines have been regarded as mechanistic and lacking agency, “a fixed response to defined stimuli” March and Simon [26], quoted in [15], p. 1); meanwhile, sociologists have come to see routines as dynamic and constantly negotiated. Participants who enact the routine are approaching it from different perspectives and may have differing goals and intentions for it. A more nuanced view of routines as suggested by the recent sociology of organizations shows that routines are not in themselves fixed responses or more bureaucratic.

Routines cannot be set aside from the people enacting them. In addition to being “repetitive, recognizable patterns of interdependent organizational actions carried out by multiple performers” [7], p. 95), routines are performed differently by different people – automatically, semi-automatically, or mindfully. Routines, even if they are habitual (like morning routines), can be meaningful for those who perform them and are not always performed without thinking. Finally, routines do not always remain stable, as studies show, there can be micro-changes that eventually lead to more change [6]. As Jennifer Howard-Grenville et al. [15] summarize, routines can be seen as effortful accomplishments that constitute agency, can be responsive to situations, and can change over time.

Hence, routines could support the decision-making process, such as the routine provision of particular information about the implications of NIPT, and routines could also allow sufficient time for reflection. Routinization of NIPT often involves various inter-connected actions whose performance can vary in terms of regularity and social control, such as: (i) the offer of a test, (ii) the testing itself, (iii) the provision of certain types of information about the implications of the test, and (iv) actions taken if the test turns out positive. These routines can be organized in better or more problematic ways, e.g. by not giving the pregnant women enough time to think about the offer or to consult others.

## Psychological perspective

As stated before, empirical studies about women’s decision making about prenatal screening report high levels of informed choice, even though more than 20% reported not to have made informed choices [52, 20]. One major problem with these studies however is the precise conceptualization of informed choice. Thus, the lack of an adequate measure of informed decision making precludes a realistic evaluation of whether or not women’s freedom of choice is under threat. Adopting a Foucauldian perspective, Mianna Meskus [27] studied women’s considerations in decision-making about prenatal diagnostic tests in highly routinized clinical contexts. She observed that selective abortion has historically been redefined from a preventive measure to a family-specific dilemma that asks the pregnant woman to develop her own life strategy and what she calls a “personalized ethics”. Freedom of choice was a prerequisite of prenatal testing as pregnant women and their partners “bear the responsibility for their choices, take care of the sick child and go through the termination of the pregnancy which always is a painful experience” (R. Salonen, quoted in [27], p. 381). Making choices about the fate of foetal life is considered a personal matter of ethical deliberation. “Women believe that they have the right and the imperative to make their ‘own’ decisions” (383). As Meskus concludes, this has “become a routine-like expectation on which the testing system relies, and to which women have grown accustomed” (385). As a consequence, women also seek the support and moral acceptance of their peers. Seeking peer support in moral judgments is however not the same thing as conformist behaviour.

This sheds a different light on routines as well. How are pregnant women referring to ‘routines’ or ‘routinization’ themselves? Routines could also, under certain circumstances, be vessels that provide helpful communicative reassurance because one is not left alone with one’s decisions. They could help to define the situation, to understand and carry the burden of responsibility and hence, as instruments of choice, provide a sense of freedom (cf. [56], p. 144).

## Experiences with the introduction of NIPT in Germany, Israel and The Netherlands

### Level and scope of routinisation and the public debate

As NIPT has a high sensitivity (> 99%) and specificity [46], from the point of view of increasing the detection rates of trisomy 21 and other genetic trisomies and conditions it would be reasonable to use NIPS universally. The American College of Medical Genetics and Genomics has recommended “informing *all* pregnant women that NIPS is the most sensitive screening option for traditionally screened aneuploidies (i.e., Patau, Edwards and Down syndromes)” [13].

Although the development, introduction and marketing of NIPT have been commercially driven by various private companies worldwide, first in the US in 2011 [29], and originally targeted the individual consumer, its advantages and popularity led to it being included into the public health system of developed countries with universal health care. NIPT entered the market mainly (but not in all countries – for the Netherlands, see below) through private providers, requiring a medical referral for high-risk pregnancy, but not yet being available in publicly funded antenatal services outside of research studies. Some countries have recently started offering (and reimbursing) the test for all pregnancies.

We chose three countries that cover a broad spectrum of NIPT implementation: (i) Germany, with its restrictive policy and a high level of moral concern about NIPT in the public discourse, much influenced by the need to break from the history of eugenics in the National Socialist era [8], (ii) the Netherlands, with its comparatively liberal public attitude despite strict regulation and with public support of NIPT [2], and (iii) Israel, somewhere between the two (for a comparison of policies governing NIPT in Germany and Israel see Foth/Nov-Klaiman, [9]). While NIPT had been widely criticized in Germany but not in Israel, Germany’s public health insurance now covers it under the condition that it is necessary to solve a serious conflict of the pregnant woman, while Israel has decided not to cover NIPT in its public “health basket”. This comparison brings together three partially overlapping sites in a multi-dimensional field that can be described using multiple comparators. Our analysis of the different policies in the three countries is organized along three key questions: 1) On what *level(s)* of social practice in prenatal diagnostics routines are established? Is it a routine offer of NIPT, together with the necessary information for individual decision-making, or is it a routine that affects the way tests are carried out as screening? 2) What is the *scope* of emerging PND routines in this country? Are they based on individualized decision-making, restricted to certain clinics or institutions, or through nation-wide programmes? 3) What are the characteristics of *public discourse* about NIPT and prenatal screening? Which concerns seem to be dominant?

We start with Germany, a country that is overall still resistant to NIPS routines, continue with Israel, where offering universal NIPS is still an experiment, and conclude with the Netherlands, where universal NIPS is offered as standard in prenatal care. Our comparison is based on analysis of policy documents concerning NIPS issued in the relevant countries, as well as on the report by Valera Lema et al. [47].

### Germany: restrictive policy and high moral concern against routines

NIPT was introduced to the German health care sector and market in 2012 by the German company LifeCodexx AG, which had been developing its *PraenaTest* in cooperation with German and Swiss PND centres and university clinics since 2009. In May 2018, six different providers of NIPT operated in the German market, with a variety of options, with NIPT available at prices of approximately EUR 200–550. NIPT was offered mainly to high-risk women upon medical indication and at their own expense, but some health insurance plans have already begun to refund NIPT on a case-by-case basis.

From its introduction, NIPT in Germany was accompanied by public controversy concerning market entry, public R&D funding and cost coverage [3, 8]. At the same time, since 2000, Germany has had a comparatively low number of Down syndrome births, with fewer than 50 children with Down syndrome per 100,000 live births (according to WHO data).^2^ Disability rights criticism was raised on constitutional, legal, political and ethical levels, and by officials as well as groups in civil society. The former Commissioner for the Disabled of the German Bundestag, Hubert Hüppe, tried in 2012 to prevent the implementation of NIPT via a legal opinion (Rechtsgutachten), which denied the legitimacy of NIPT, mainly based on the argument that it is of no medical use and discriminates against disabled persons [12]. “Pro-life” (Lebensschutz) groups, as well as Christian care providers, organised regular demonstrations in front of the LifeCodexx headquarters in Konstanz.^3^ Following medico-technological assessment initiated by the German government in 2016^4^ of whether NIPT should be covered by the mandatory health care services, 20 German disability advocacy organizations and networks protested, claiming that NIPT had no therapeutic benefits and did not improve the medical care of the pregnant woman or the expected child.^5^ The *Arbeitskreis Frauengesundheit* wrote: “the prenatal search for genetic characteristics is not pregnancy screening, but a selective search for unwanted deviations.”^6^ These criticisms were echoed, together with supporting voices, in a heated parliamentary debate on 11 April 2019.

Against this backdrop, in September 2019, the Federal Joint Committee (Gemeinsamer Bundesausschuss, G-BA) for public health decided that NIPT should be covered, in *justified individual* cases and after medical consultation.^7^ After a public consultation organized by the G-BA in April 2021 on the draft of an unbiased information leaflet about NIPT for women,^8^ the approved version was published in November 2021. Its preface declares that NIPT “does *not* belong to the generally recommended examinations”^9^ in pregnancy care (our translation, emphasis in the original, p. 2). The text of the G-BA decision of 2019 stresses that NIPT should not be used routinely or as screening; the aim of the decision is to avoid invasive examinations and their related risk of miscarriage as far as possible.^10^ The wording of the decision explicitly avoids using a cut-off risk value for Down syndrome as an indication for NIPT. All decisions about offering or using NIPT should be on a case by case basis, only if it is necessary because of the “personal situation” of the woman who is “so heavily burdened by the possibility of a trisomy that she wants to get it checked” (leaflet, p. 11; our translation). This means that NIPT in Germany will mainly stay a second-tier test, but is covered by health insurance and should be offered after detailed counselling to women who feel burdened by the risk on a case-by-case basis.

In the German public discourse on NIPT, the word “routinization” is used negatively to indicate a fear of social pressure, a selective, eugenic attitude and a challenge to individual autonomy. This is illustrated by the wording of the invitation to a public online forum on NIPT, organized by the German Ethics Council on 23 February 2022, which addressed “the fear” that it “could become routine practice”.^11^

### Israel: Individual choice for a technically superior test.

As in Germany, NIPT is offered in Israel by obstetricians and geneticists to pregnant women who are at risk of fetal chromosomal abnormalities due to age or family history. In a position paper from 2013, the Israeli Association of Geneticists [28] recommended that NIPT should *not* replace existing screening, namely first trimester ultrasound for nuchal translucency and biochemical tests. Although NIPT is currently not part of the Israeli ‘health basket’ (meaning that its cost is not covered by the Ministry of Health), various financial agreements exist between the seven private companies offering NIPT in Israel and Israeli HMOs (Health Management Organizations providing health insurance). These agreements, as well as the specific insurance the woman holds, determine the fraction of the test cost paid by the HMO and the fraction paid by the woman. The overall cost of NIPT is relatively high: around NIS 3000–4000 (approx. EUR 700–1000,[35].

However, in 2019 the Israeli Association of Geneticists voted to replace the current biochemical screening with NIPS, and NIPS was submitted to the “health basket” committee for possible inclusion in 2020, where it competed against other technologies and drugs in the context of a limited budget and was rejected. Consequently, NIPT is not covered by statutory health insurance. Israeli women are currently offered this test privately (or semi-privately, as most complementary health insurance policies cover 75% of the cost). A recent unpublished report of the Israeli Ministry of Health on uptake in 2014–2017 (personal communication with A. Singer, Head of the Dept. of Community Genetics at the Israeli MoH) showed a high uptake of invasive prenatal tests (11.8% out of Israeli women who gave birth that year), while NIPS was only performed in 4.3%. NIPS uptake did not rise significantly in these years, alongside a very modest decline in invasive procedures. In contrast to the rapid decrease in the rate of invasive testing in the “NIPS era” observed in many other countries, Israeli women seem generally to prefer the more diagnostically accurate and comprehensive chromosomal microarray analysis (CMA) testing, which in Israel is integrated into amniocentesis when medically indicated [43].

There has been no public debate about NIPT/NIPS in Israeli public media. The chairperson of one of Israel’s Down syndrome advocacy organizations has called upon the Israeli government to subsidize NIPT and make it available to all women that “the cost of this test is an outrageous wrongdoing… Since it is a blood test (a short and simple procedure) and since this test could prevent, for those who wish it, bringing into the world a child with special needs, it seems to us extremely important to reduce the costs of the test immediately and make it accessible.”^12^ According to the WHO, the number of DS births in Israel had dropped from 114 in 100,000 in 1980 to 68 in 2015^13^; still higher than in Germany.

In 2019, for the first time and with the approval of the Ministry of Health, a major Israeli hospital in Tel Aviv started offering NIPS to all pregnant women from week 8 of pregnancy, through the hospital’s Genetics Lab, for a reduced cost of NIS 2600 (approx. EUR 650). Following standard practice in Israel the new service includes pre- and post-test-counselling. This local service may soon pave the way to further routinization in the universal offer of NIPS.

### The Netherlands: strict regulation of procedures and liberal public attitude

In Belgium, Denmark and the Netherlands, NIPS is currently offered to all pregnant women as first-tier screening [47]: 52). From 2014, in the context of the research study Trident 1, NIPS was recommended by the Health Council of the Netherlands^14^ as a first-line screening test for T21, T18 and T13 instead of the combined test for women at increased risk^15^ In 2017, the Dutch government started a follow-up Trident 2 research study offering a choice between FCT (first trimester combined test) and NIPT to all pregnant women. Women pay EUR 175 for a first-tier NIPT, which is similar to the cost of FCT (approx. EUR 168). NIPS is subsidized by the Dutch government. The media described the new choice enthusiastically, exclaiming that hospitals “expect a rush of pregnant women”.^16^ Analysis of the questions asked by Dutch women and health professionals on national health websites during 2013–2015 (the time of the Trident study) showed that most concerned the conditions and population eligible for NIPT, suggesting public interest in a broader scope for the test [45]. Studies of the views of women about their experiences of decision-making about NIPT in the Netherlands show high rates of informed decision-making (using a specific measure) and perceived freedom [49, 50]. Moreover, in a qualitative study by Garcia et al. [11] all 29 pregnant women who were offered NIPT stated that they made their own decision freely without being constrained by societal expectations, while some participants speculated that societal pressure on expectant mothers toward the use of screening and termination of an affected pregnancy could increase because of the improved characteristic of NIPT. They did not feel moral pressure and they do not see NIPT as an obligation of responsible motherhood [11].

However, the routinization of the *offer* does not imply high *uptake* in this case. The current uptake of NIPS is about 40–45% of pregnant women, and did not change significantly between 2003 and 2016 [48].^17^ Participation in the combination test decreased sharply due to the introduction of NIPT: from 34.1% in 2016 and 12.4% in 2017 to 2.5% in 2018 and 1.7% in 2019 [21]. Nevertheless, this represents an increase when compared to the even lower uptake of conventional screening tests for DS (< 30%) reported in the Netherlands – especially when compared to an FCT uptake of 74% in England, 84% in France and > 90% in Denmark [5]. Pre-test counselling and information leaflets about NIPS emphasize that it should be the pregnant woman’s own informed decision to accept the test offer or not, with an emphasis on the right not to know.^18^ This is in line with the WHO data that the Netherlands have a relatively high incidence of births with Down syndrome, which has grown from about 100 per 100 000 live births in 1996 to about 200 in 2015, mostly due to an increased age of the mother.^19^

In the Dutch case [51, 53], the strictly regulated routine universal offer of NIPS is seen by policy-makers as the best way to safeguard women’s choices. As prenatal screening falls under the Population Screening Act, a routine offering of universal NIPS allows the Minister to set quality requirements and licensing for providers, including counselling training and educational materials. The Public/Population Screening Act (Wet Bevolkingsonderzoek, WBO) is intended to protect individuals against screening that may pose a medical danger, for example through radiation (e.g. screening for cancer), or if there is no treatment for a condition (and abortion is not considered to be a treatment). For example, the nationwide Dutch prenatal screening programme, supported by the WBO, not only provides standards for regional and nationwide coordination and quality assessment of prenatal screening but also defines how the offer of screening is to be routinized.

### Comparison of the policies in Germany, Israel and the Netherlands: level and scope of routinization, and public debate

While the situation is still dynamic, Germany, Israel and the Netherlands highlight important axes in the process of routinization, such as the level of routinization (offering, information and counselling, use, how to deal with the test results); the scope of routinization (national or local); and the public discourse surrounding the meanings of routinization. A comparison like this shows how national variations exist alongside co-evolution in terms of the stages and goals of the process of routinization (see Table 1).

**Table 1:** Comparing Germany, Israel and the Netherlands in terms of NIPT routinization

**Country**	Germany	Israel	The Netherlands
**Axes of Routinization**			
Level of routinization of offering NIPT and providing information	Against routinization of a universal offer – must be offered on a case-by-case basis	Ongoing routinization of universal offer at specific hospitals	Universal offer is strictly regulated under a specific law (with a traditionally low uptake rate of DS screening)
Scope of routinization	Limited to the conditions in individual cases	Local routines at specific hospitals	Nationwide as a first-tier screening test
Public discourse	Objection to the routinization of screening as eugenic	No public discourse in the media about NIPT routinization	Supportive of NIPT routinization; positive attitude towards test and firm belief in free and informed choice by pregnant women

While a routine universal offering of NIPT may increase the uptake rate simply due to greater accessibility, this also depends on previous tendencies and cultural circumstances. In a traditionally test-critical environment such as the Netherlands, the routine universal offer increases an uptake that nevertheless remains at a relatively low level. In a pro-test environment such as Israel [39], a routine universal offer of NIPT would probably lead to a very high uptake, as many Israeli women currently refrain from having NIPT because of its high cost, and as such may lead to inequitable access to prenatal screening. Once NIPT’s cost is covered by the health insurance for all pregnant women, many women would probably have NIPT first (as this can be done very early) and then go on to have the medically required, more detailed and diagnostic amniocentesis, often together with CMA. The German example shows that even a limited offer is fraught with concerns about the medical and/or social expectations of a test that is paid for by health insurance, and how it should be offered. How this will be enacted in practice however remains to be seen. Anecdotical evidence shows that insurance coverage of NIPT is understood at least by some women as meaning that NIPT is a good thing to do.^20^ These concerns and attitudes however may actually hinder the implementation of prenatal screening and the NIPT based on explicit quality criteria and may lead to a lower quality screening routine, especially in relation to informed choice. It is intriguing that routines of universal screening can be seen as both the source of a problem and its antidote, depending on the country and the kind of routines established. Thus, in the Netherlands, from the beginning there was a strong emphasis on informed decision-making secured through routinization, for example by mandating that only trained and licensed healthcare providers should be allowed to offer NIPT. On the other hand, the pragmatic approach of the Netherlands may also distract from the inherently complex ethical considerations associated with prenatal testing.

We focused on the ethical aspects involved in the actual practice of routines, which remain largely hidden from many technological assessments^21^ and are overlooked in many ethical analyses that are critical of routinization per se. Our comparison of the three countries shows that routinization does not necessarily antagonise autonomous decision-making and choice. In the Netherlands, there are high rates of informed decision-making and perceived freedom to choose in cases of foetal aneuploidy screening, suggesting that there is little reason for concern about routinization of NIPT based on the perspectives of pregnant women [49, 11]. Whether people may accept or reject NIPT depends on many factors, including the nature and scope of the offer and how it is routinized, and how the situations of individual decision-making are (routinely) cultivated, as well as on broader social tendencies and personal considerations.

Kater-Kuipers et al.’s [19] list of potential concerns about routinization can be used to drive an empirical, socio-ethical examination of routinization-in-practice. *If* the procedures hinder informed choice, *if* they put social pressure on women, and/or *if* they lead to a neglect of disability rights, they are indeed problematic. Responsible implementation of NIPT, e.g. within a national screening programme, is however a prerequisite, for a high quality program in all aspects including informed choice.

Hence, the critiques can be read as evaluation criteria for checking routines and – if necessary – for changing them. In many of the examples we described, specific interventions or supportive actions to provide balanced information and counselling were taken to respect the pregnant woman’s autonomy when the technology is used. The universal routinization of NIPS as the better test for all pregnant women is justified in the Dutch case by the extra protection offered through the Population Screening Act (WBO) and its mandatory certified licensing and training. In the Netherlands there are also concerns about routinization, but universal routinization of offering screening is predominantly seen as a way to protect individual decision-making by pregnant women. As the Dutch experience shows, routinization of offering NIPT, under appropriate conditions, does not lead to devaluing free and informed decision-making by individuals. But for this to be the case, a routinized offer of a test must be accompanied by other parts of the routines that provide relevant information and support for personal decision-making.

## Conclusions

Routinization in prenatal diagnostic practice raises ethical questions, but routinization per se cannot be ethically evaluated without greater specification from a sociological and psychological perspective. Some forms of routines can be empowering and endorse agency, while others can hamper free and informed decision-making. It also depends on a positive or negative image of people with Down Syndrome, availability of support and on their inclusion in society. Strict regulation and responsible implementation of NIPT can safeguard quality of care as well as women’s autonomy. However, whether this is the case for all women remains to be shown, as in the Netherlands where the uptake of the NIPT is much lower in socioeconomically disadvantaged neighbourhoods [50], signalling the need for an approach that guarantees equal access.

Open questions remain to be answered by empirical follow-up studies in different countries. Three groups of issues should be emphasized. First, since in most cases prenatal diagnosis does not lead to treatment or prevention other than abortion, it remains to be seen whether, how far and under which contextual conditions the argument that individual informed choice is routinely enabled, can convincingly outweigh concerns about selective reproduction. Thus, the discussion about the inherent complex ethical considerations related to prenatal screening decisions remains pertinent. Second, some aspects of the setting may contribute to the acceptability of NIPT routines to disability advocates. In a non-inclusive societal environment the existence of a routine offer of NIPT, even though it remains strictly on the basis of individual free decision-making, can be perceived as sending a difficult message for those living with the conditions for which tests are offered. And, most importantly, it depends on how children with disabilities are included and supported (instead of discriminated against) in society [33, 38].

Third, pregnant women have different vulnerabilities. Some forms of (relational) autonomy are worth having [18], other ideas about and forms of (individualized) autonomy are more of a burden and serve to displace responsibility onto the pregnant woman alone [27, 36]. Decision-makers in such existentially sensitive areas need not only information but also good relationships and communicative reassurance, in order to make acceptable decisions that can be trusted in retrospect. It cannot be wrong per se to seek confirmation in what others do and how they reason for it, and therefore to strive for a certain conformity with what one sees as justified, as long as the decision taken is well considered.

Routinization of screening can be seen both as the source of a problem and also its antidote, however dependent on the level and content of routines, which in our view suggests a need to open up the concept of routinization and look for an interdisciplinary definition of its meanings and elements. While an ethical critique on routinization of NIPT is valid, it needs to be complemented by sociological and a psychological perspectives related to evidence on actual informed choice of pregnant women and the broader sociological context of quality and access to care. Our analysis confirms Ruth Horn’s proposition that the ethical acceptability of routines depends on the availability of information and social support to raise a child with a disability [14]. If women within the routines have the necessary space for decision-making they may find it possible, even easy, to make decisions about NIPT in line with their values and independently of others’ opinions.

The discussion about routinization in the field of prenatal diagnostics (and also in healthcare beyond it) should incorporate and work with the necessary distinctions between levels and forms of routines, in order to develop sound criteria for their ethical evaluation. Routinization per se is too blunt a term to be discussed ethically. It needs to be empirically established whether or not routinization affect individual informed decision-making and restrict freedom of choice due to perceived social expectations to act in a certain way. Furthermore, whether or not prenatal screening has a negative impact on people with disability, need to be discussed in light of the inclusiveness (or not) of a society. Routine abortion for conditions like Down syndrome would be considered the ethically most problematic level of routinization, if the termination is not well considered individually but made ‘by the book’. Yet the choice about the child women do or do not want to give birth to reflects parental autonomy as long as it is their choice. Routines that are unavoidably emerging as part of social and health care practice should therefore have built-in checks and balances to ensure that communication is two-way, information is balanced, and choice really is informed and well-considered. Moreover, all these elements should be assessed in the context of wider society as well, and include the interests of people with disabilities.

An ethics of routines should not just focus on discussing the normative statements contained in regulations and guidelines. It needs to consider the development of routines in the context of emerging social practices and individual informed choices.

## Data Availability

N.a.

## Footnotes

1 A point anticipated by Barbara Katz Rothman long before the advent of NIPT: “When risk is removed, the last completely socially acceptable reason for not wanting to know fetal defects will be gone” [40], p. 82).

2 See WHO European Health Information Gateway, Births with Down's syndrome per 100 000 live births: https://gateway.euro.who.int/en/indicators/hfa_603-7120-births-with-downs-syndrome-per-100-000-live-births/visualizations/#id=19698 [27.07.2023].

3 See the invitation note from the CDL (Christdemokraten für das Leben), an initiative of the Christian Democratic Party CDU: Herzliche Einladung zur Demo gegen den PraenaTest am 16. Juli 2017 in Konstanz, https://cdl-online.net/herzliche-einladung-zur-demo-gegen-den-praenatest-am-16-juli-2017-in-konstanz/543 [27.07.2023]: “protestieren Sie so gegen den neuen, bedrohlichen Gentests (PraenaTest), der die vorgeburtliche Selektion von Menschen mit Behinderungen zum Ziel hat!” (protest against the new, dangerous gene tests (PraenaTest), the aim of which is to select prenatally against people with disabilities!).

4 Cf. Gemeinsamer Bundesausschuss (G-BA) (2016): Pressemitteilung Nr. 32/2016: Methodenbewertung. Nicht-invasive Pränataldiagnostik bei Risikoschwangerschaften – G-BA beginnt Verfahren zur Methodenbewertung – Beratungen zur Erprobung ruhend gestellt, Berlin, 18.08.2016, https://www.g-ba.de/downloads/34-215-635/32_2016-08-18_Methodenbewertung%20NIPD.pdf [27.07.2023].

5 Stellungnahme: Keine Aufnahme weiterer selektiver Untersuchungen in die Regelversorgung! (14 February 2017), https://www.arbeitskreis-frauengesundheit.de/2017/02/14/stellungnahme-keine-aufnahme-weiterer-selektiver-untersuchungen-in-die-regelversorgung/ [27.07.2023].

6 Our translation. “Die pränatale Suche nach genetischen Merkmalen ist eben keine Schwangerenvorsorgeuntersuchung, sondern eine selektive Fahndung nach unerwünschten Abweichungen.” https://www.arbeitskreis-frauengesundheit.de/2017/02/14/stellungnahme-keine-aufnahme-weiterer-selektiver-untersuchungen-in-die-regelversorgung/ [27.07.2023].

7 For a further analysis of how the G-BA and other German governance actors addressed the situation of fragmented responsibility in relation to NIPT up to 2017, see Braun & Könninger [3],an ethical analysis of the decision is provided in Rehmann-Sutter/Schües [37].

8 Gemeinsamer Bundesausschuss: Pressemitteilung 15 April 2021: Versicherteninformation zum nichtinvasiven Pränataltest auf Trisomien: G-BA leitet breites Stellungnahmeverfahren ein. https://www.g-ba.de/presse/pressemitteilungen-meldungen/952/ [27.07.2023].

9 Gemeinsamer Bundesausschuss: Bluttest auf Trisomien. Eine Versicherteninformation (Nov. 2021), p. 2. https://www.g-ba.de/downloads/17-98-5156/2021-11-09_G-BA_Versicherteninformation_NIPT_bf.pdf [27.07.2023].

10 G-BA Pressemitteilung „Nicht-invasiver Test zum Vorliegen von Trisomien als mögliche Alternative zu invasivem Eingriff “: https://www.g-ba.de/presse/pressemitteilungen/810/ [27.07.2023].

11 Bioethics Forum: Good to Know? On the Responsible Use of Non-Invasive Prenatal Testing. 23 February 2022. https://www.ethikrat.org/en/bioethics-forum/good-to-know/?cookieLevel=not-set&cHash=4632649900bfbe9d87f314ce43bd8294 [27.07.2023].

12 Personal communication between the third author and Rachel Lishansky, chairperson of ATID Israeli association for DS advocacy, Dec. 10, 2019.

13 https://gateway.euro.who.int/en/indicators/hfa_603-7120-births-with-downs-syndrome-per-100-000-live-births/visualizations/#id=19698 [27.07.2023].

14 Health Council of the Netherlands. Population Screening Act: noninvasive prenatal test for increased risk of trisomy. The Hague: Health Council of the Netherlands, 2013; publication no. 2013/35. https://www.gezondheidsraad.nl/documenten/adviezen/2013/12/17/bevolkingsonderzoek-niet-invasieve-prenatale-test-bij-verhoogd-risico-op-trisomie [27.07.2023].

15 NIPT Consortium, The Netherlands https://niptconsortium.nl/english/ [27.07.2023].

16 Pieters, J. Dutch Hospitals Expect Rush on NIP-Test, NLTimes, 30 March 2017, https://nltimes.nl/2017/03/30/dutch-hospitals-expect-rush-nip-test-belgians-sue-nl-illegal-state-aid-test [27.07.2023].

17 Melanie Zierse: NIPT of niet, zwangeren hoeven niet alles van tevoren te weten. Trouw 22 September 2017. https://www.trouw.nl/nieuws/nipt-of-niet-zwangeren-hoeven-niet-alles-van-tevoren-te-weten~b89a6c51/ [27.07.2023]. For numbers of participation for prenatal screening see: https://www.staatvenz.nl/kerncijfers/zwangerschapsscreening-down-edwards-pataussyndroom-deelname [27.07.2023].

18 See Rijksinstituut voor Volksgezondheid: The NIPT, testing for Down’s, Edwards’ and Patau’s syndromes. https://www.pns.nl/documenten/information-about-prenatal-screening-for-down-syndrome-edwards-syndrome-and-pataus [27.07.2023].

19 VZinfo, Downsyndroom, https://www.volksgezondheidenzorg.info/onderwerp/downsyndroom/cijfers-context/trends#node-trend-geboorteprevalentie-downsyndroom [27.07.2023].

20 Anke Hartmann (ongoing medical dissertation).

21 As is the case with the “rapid” procedure, which was used in Valera-Lema et al. (47), it did not include ethical analysis, in contrast to the full version of HTA according to the Core Model, which would include ethical analysis.
